# Lactate regulates pathological cardiac hypertrophy via histone lactylation modification

**DOI:** 10.1111/jcmm.70022

**Published:** 2024-08-28

**Authors:** Shuai‐Shuai Zhao, Jinlong Liu, Qi‐Cai Wu, Xue‐Liang Zhou

**Affiliations:** ^1^ Department of Cardiac Surgery, The First Affiliated Hospital, Jiangxi Medical College Nanchang University Nanchang China; ^2^ Institute of Translational Medicine Shanghai University Shanghai China

**Keywords:** cardiac hypertrophy, H3K18la, histone lysine lactylation, HKla, post‐translational modification

## Abstract

Under the long‐term pressure overload stimulation, the heart experiences embryonic gene activation, leading to myocardial hypertrophy and ventricular remodelling, which can ultimately result in the development of heart failure. Identifying effective therapeutic targets is crucial for the prevention and treatment of myocardial hypertrophy. Histone lysine lactylation (HKla) is a novel post‐translational modification that connects cellular metabolism with epigenetic regulation. However, the specific role of HKla in pathological cardiac hypertrophy remains unclear. Our study aims to investigate whether HKla modification plays a pathogenic role in the development of cardiac hypertrophy. The results demonstrate significant expression of HKla in cardiomyocytes derived from an animal model of cardiac hypertrophy induced by transverse aortic constriction surgery, and in neonatal mouse cardiomyocytes stimulated by Ang II. Furthermore, research indicates that HKla is influenced by glucose metabolism and lactate generation, exhibiting significant phenotypic variability in response to various environmental stimuli. In vitro experiments reveal that exogenous lactate and glucose can upregulate the expression of HKla and promote cardiac hypertrophy. Conversely, inhibition of lactate production using glycolysis inhibitor (2‐DG), LDH inhibitor (oxamate) and LDHA inhibitor (GNE‐140) reduces HKla levels and inhibits the development of cardiac hypertrophy. Collectively, these findings establish a pivotal role for H3K18la in pathological cardiac hypertrophy, offering a novel target for the treatment of this condition.

## 1 INTRODUCTION

Pathological cardiac hypertrophy refers to the enlargement of cardiac mass and volume caused by various factors, including hypertrophy of cardiomyocytes, proliferation of myocardial interstitial cells and changes in the myocardial extracellular matrix.^1^ Meanwhile, cardiac hypertrophy increases the demand for oxygen in the myocardium. This increase in oxygen consumption leads to a mismatch in coronary blood supply and demand, resulting in the aggravation of myocardial ischaemia. Ischaemia and hypoxia, in turn, further aggravate myocardial hypertrophy.^2^ Cardiac hypertrophy is an adaptive response of cardiomyocytes to increased pressure load. Mechanically, the adaptive hypertrophic response is initially triggered by stress stimulation, which produces enough force to resist the elevated wall tension or pressure/volume overload in cardiomyocytes and maintain normal blood circulation in the heart. Prolonged pressure load stimulates the heart, resulting in ventricular remodelling and cardiac dysfunction, ultimately leading to heart failure (HF).^3^ The remote regulation of the microenvironment following ventricular remodelling is highly complex and influenced by multiple factors.

Glucose and oxygen are the primary substrates for cell metabolism. In cases of cardiac hypertrophy caused by pressure overload, cardiomyocytes utilize glucose as their primary substrate due to its high energy utilization rate.^4^ One molecule of glucose produces two molecules of ATP and lactate. ATP production is crucial for maintaining the contractile function of a healthy heart. Under hypoxic conditions, cells stimulate lactate production by inhibiting oxidative phosphorylation and enhancing glycolysis.^5^ Lactate plays an important role in epigenetic modification and metabolic reprogramming by regulating energy metabolism, immune response and cell‐to‐cell communication.^6^, ^7^ It is common for local lactate accumulation to be caused by an acute inflammatory response or inadequate perfusion.^8^ Cardiac hypertrophy, which inhibits oxidative phosphorylation and reduces mitochondrial energy production, impairs overall cardiac efficiency. This creates conditions favourable for the progression of HF.^9^

Numerous studies have demonstrated the significance of epigenetic alterations in regulating cell proliferation, protein synthesis and gene transcription, including N6‐methyladenosine (m^6^A) modification, phosphorylation, acetylation and ubiquitination.^10^ The interaction between epigenetics and metabolism is crucial in controlling gene expression, cell proliferation and cell differentiation.^11^ In the past, lactate was considered a metabolic waste product of glycolysis, a result of the Warburg effect. However, lactate is now recognized as a novel source of energy, a signalling molecule and an immunoregulatory molecule. It also plays a crucial role in regulating gene expression through a type of histone modification known as histone lysine lactylation (Hkla).^12^ In 2019, HKla was newly characterized as an epigenetic modification, and its presence in mammalian cell lines was confirmed through mass spectrometry. Lactate serves as a substrate for lactyl‐CoA, which regulates gene expression by lysine lactylation on histones in various pathophysiological conditions.^13^

The post‐translational modification (PTM) state of core histones dictates whether chromatin is suppressed or activated for transcription and translation.^14^ Lactylated proteins are widely involved in PTM, genetic recombination and cellular metabolism.^15^ Studies on gene transcriptional regulation have shown that PTM is one of the key mechanisms in regulating cardiac hypertrophy.^16^ Previous studies have demonstrated that hypoxia‐induced elevation of intracellular lactate levels promotes HKla expression, leading to the reprogramming of cellular metabolism.^13^ This study investigated the expression of HKla in neonatal mouse cardiomyocytes (NMCMs) stimulated with Ang II and in an animal model of cardiac hypertrophy induced through transverse aortic constriction (TAC) surgery.^17^ Additionally, we explored the dynamic patterns of HKla during cardiac hypertrophy. We analysed the dynamic changes of H3K18la, H3K9la, H4K5la, H4K12la, H3K14la and Pan Kla using western blot and compared them with H3K18ac, H4K5ac and Pan Kac. In this study, we provide new insight into pathological cardiac hypertrophy mechanisms involving metabolomes and epigenomes.

Given the improvement in living standards and the ageing population, the incidence and prevalence of pathological cardiac hypertrophy and HF have significantly increased. Nonetheless, the pathomechanism of pathological cardiac hypertrophy remains unclear, and the clinical treatment effect on myocardial hypertrophy and HF is suboptimal. Therefore, there is an urgent need for a comprehensive investigation into the pathomechanism of myocardial hypertrophy and the identification of new biomarkers for diagnosis and treatment.

## 2 MATERIALS AND METHODS

### 2.1 Ethics Statement

This study was conducted with the approval of the Ethics Committee of the Medical College of Nanchang University. The experimental protocols were in accordance with the Animal Care Guidelines of the National Institutes of Health.

### 2.2 Reagents

Pan anti‐Kla, anti‐H3K18la, anti‐H3K9la, anti‐H3K14la, anti‐H4K5la and anti‐H4K12la antibodies were purchased from Genelily Biotech (Shanghai, China). Pan anti‐Kac antibody was purchased from CST (USA). Anti‐ANP antibody was purchased from Proteintech (Wuhan, China). Anti‐histone H3, anti‐H3K18ac, anti‐H4K5ac, anti‐LDHA, anti‐LDHB, anti‐p300, anti‐β‐MHC, anti‐BNP and anti‐GAPDH antibodies were purchased from Abclonal Biotech (Shanghai, China). Reagents including glucose, lactate, 2‐Deoxy‐d‐glucose (2‐DG), oxamate and GNE‐140 were purchased from MedChemExpress (Shanghai, China). Anti‐fibronectin, anti‐α‐SMA, anti‐collagen I, anti‐tensin and antibodies were purchased from Abcam (Shanghai, China). Goat anti‐rabbit IgG H&L (HRP) and goat anti‐mouse IgG H&L (HRP) secondary antibodies were purchased from Abclonal Biotech (Shanghai, China).

### 2.3 Animal experimental protocols

Male C57BL/6 mice (8 weeks old; weighing 20–23 g) used in this study were purchased from the Cavens Biogle (Suzhou, Jiangsu, China) Model Animal Research Co. Ltd. The animals were housed in temperature‐controlled cages with a 12‐h light/dark cycle, and were provided with free access to food and water. C57BL/6 mice were anaesthetised using intraperitoneal injection of pentobarbital sodium at a dose of 50 mg/kg.^17^ After the toe pinch reflex disappeared, the mice were fixed in the supine position and connected by laryngotracheal intubation to a small animal ventilator. The aortic arch was exposed through a median anterior chest incision, a 27‐G needle was inserted between the brachiocephalic artery and the left common carotid artery. Next, ligated with 6‐0 silk between the brachiocephalic artery and the left common carotid artery. After the ligation procedure, the needle was promptly removed, resulting in an approximately 75% reduction in the cross‐sectional area of the aortic arch. The mice in the sham surgery group underwent the same procedure, except that their aorta was not constricted. Pleural fluid was drained with a sterile cotton ball, pleural gas was evacuated, and the chest wall and skin were sutured. After surgery, the mice were placed on a 37°C heating pad for observation until recovery. Subsequently, the mice were randomly allocated into two groups: the TAC group and the sham group, and continued to be fed for 28 days (*n* = 6 mice/group).

### 2.4 Echocardiography of mice

Four weeks after surgery, cardiac function was assessed in all mice using a blinded method. C57BL/6 mice were anaesthetised using intraperitoneal injection of pentobarbital sodium at a dose of 50 mg/kg. After the toe pinch reflex disappeared, the mice were fixed in the supine position. Echocardiography was performed using the Vevo 2100 imaging system (FUJIFILM Visualsonics, Toronto, Canada). The left ventricular end‐diastolic diameter (LVEDD), left ventricular posterior wall thickness (LVPWT) and left ventricular end‐systolic diameter (LVESD) were measured in the long‐axis view of the left ventricle. The left ventricular ejection fraction (LVEF) and left ventricular fractional shortening (LVFS) were measured using M‐mode ultrasound. After echocardiography, the mice were euthanized by CO_2_ inhalation. The tissues were isolated for the histological analysis.

### 2.5 Histological analyses

After completion of the cardiac function test, the heart was quickly extracted by thoracotomy, and subsequently, the pericardial tissue and blood vessels were eliminated. The weight of each heart was obtained using an electronic balance. Calculate heart weight/body weight (HW/BW); heart weight/tibia length (HW/TL).

### 2.6 Haematoxylin and eosin staining

The left ventricular tissue of the mouse heart was fixed in 10% formalin and embedded in paraffin using standard histological procedures. Serial 5‐μm‐thick sections were obtained. The paraffin sections were deparaffinized, stained with haematoxylin and eosin and sealed with neutral gum for preservation. Observations were obtained by microscope (Olympus, CKX53) and high‐quality images were captured for subsequent analysis.

### 2.7 Wheat germ agglutinin (WGA) staining

The hearts were isolated and fixed in 10% formalin using standard histological protocols, followed by embedding in paraffin. Subsequently, sections with a thickness of 5 μm were prepared. The paraffin sections were deparaffinized by rinsing them with xylene, 75% alcohol and distilled water. Then, 1:1000 WGA (Thermo Fisher, USA) was added and the samples were incubated for 1 h at room temperature in a wet box. Sections were labelled with DAPI (1 μg/mL, Thermo Fisher, USA) fluorescein and incubated at room temperature for 30 min, followed by washing with PBS. Finally, the samples were sealed with a water‐soluble sealer. Histological changes were observed and recorded using an inverted fluorescence microscope (IX81; Olympus). The cross‐sectional area of cardiac myocytes was calculated by measuring the areas of different fluorescent regions using Image‐ProPlus_6.0 software.

### 2.8 Masson's trichrome staining

Paraffin‐embedded myocardial sections were prepared, followed by routine deparaffinization. The sections were stained with iron haematoxylin (Beyotime, China) and Ponceau (Beyotime, China), treated with phosphomolybdic acid (Beyotime, China), stained with aniline blue (Beyotime, China), dehydrated, fixed and sealed with rubber. Histological changes were observed and recorded using a microscope, and high‐quality images were captured for subsequent analysis. Five random fields were selected in each section to measure the fraction of myocardial collagen area. To ensure a comprehensive analysis, 5 slices per animal were selected randomly yet systematically for further detailed examination. The collagen area fraction was calculated using the formula % area collagen = (area of collagen/area of full field of view) × 100%.

### 2.9 Culture and modelling of neonatal mouse cardiomyocytes

Hearts were isolated from neonatal C57BL/6 mice at 1–2 days of age and washed three times with d‐hanks solution. The experimental protocols were conducted with the approval of the Ethics Committee of the Medical College of Nanchang University and in accordance with the Animal Care Guidelines of the National Institutes of Health. The apical LV myocardium was excised, cut to 1 mm^3^, and digested with 0.03% trypsin and 0.04% collagenase II (MCE, Shanghai). Subsequently, the cells were transferred to DMEM (Gibco, Thermo Fisher, USA) medium supplemented with 10% fetal bovine serum. After filtration, the cell suspension was prepared and cardiomyocytes were separated by the differential adhesion method according to the different adhesion rates of cardiomyocytes and fibroblasts. In the inoculation culture, 0.1 mM BrdU (Thermo Fisher, USA) was added for the first 3 days to inhibit fibroblast growth. After removing the cell suspension by aspiration, the NMCMs were transferred to complete medium and incubated in a 37°C incubator with 5% CO_2_ for 24 h. Finally, the cardiomyocyte hypertrophy model was established by stimulating NMCMs with 1 μM Ang II (MCE, China) for 24 h.^18^

### 2.10 Measurement of cardiomyocyte surface area

We performed immunofluorescence staining to measure cardiomyocyte surface area as previously described.^19^ NMCMs were treated with 4% paraformaldehyde for a duration of 20 min, followed by washing using PBS with 0.1% Triton X‐100. Subsequently, cells were incubated with anti‐α‐actinin antibody (1:400, Cat. No. ab90421, Abcam, China) overnight at 4°C. Incubation with goat anti‐mouse IgG H&L (Alexa Fluor® 488) secondary antibody (1:2000, Cat. no: ab150113, Abcam, China) was continued for 2 h at room temperature, and nuclei were labelled with DAPI staining for 10 min. Afterwards, the cells were observed and recorded with a confocal fluorescence microscope (LSM800; Carl Zeiss, Oberkochen, Germany). The cell size was quantified utilizing Image‐Pro Plus 6.0.

### 2.11 Lactate assay

Intracellular levels of lactate were measured by employing colorimetric L‐lactate assay kits (Solarbio, Beijing, China) following the guidelines provided by the manufacturer. The spectrophotometric microplate reader was used to measure absorbance at a wavelength of 570 nm. The concentration of lactate within samples was calculated by comparing the sample optical density to the standard curve.^15^

### 2.12 Seahorse ECAR analysis

To investigate mitochondrial bioenergetics in cellular preparations, we utilized the Seahorse XF24 instrument (Agilent Technologies, Santa Clara, CA, USA). The experiment was conducted as follows: The primary neonatal mouse cardiomyocytes (10^4^/well) were seeded in XF24‐well culture plates and allowed to adhere for 24 h at a confluence of 70%. The cells were then treated according to the experimental design. Before the assay, cells were washed twice with XF base medium without sodium bicarbonate, adjusted to pH 7.4, supplemented with 10 mM glucose and incubated in a non‐CO_2_ incubator at 37°C for 1 h. During the assay, a glycolysis test kit was performed by sequentially injecting compounds affecting glycolysis (glucose, oligomycin and 2‐DG) while measuring extracellular acidification rate (ECAR) using the Seahorse XF24 Analyzer. Data were analysed using Wave software (Agilent Technologies).^20^

### 2.13 Western blotting

The histones were extracted from the hearts of C57BL/6 mice and NMCMs following previously described methods.^17^, ^21^ Target proteins were transferred to a PVDF membrane (MerckMillipore, USA) after being subjected to 10% SDS–PAGE. Afterwards, the membrane was obstructed using 5% skim milk at a temperature of 25°C for a duration of 1 h. This was then followed by an overnight incubation with primary antibodies against ANP (1:1000, 27426‐1‐AP, Proteintech, China), BNP (1:1000, A2179, Abclonal, China), β‐MHC (1:2000, A7564, Abclonal, China), LDHA (1:1500, A1146, Abclonal, China), LDHB (1:1500, A7625, Abclonal, China), P300 (1:1000, A1324, Abclonal, China), GAPDH (1:6000, AC001, Abclonal, China), Lactyl‐Histone Antibody Sampler Kit (1:1000, PTM‐7093, PTM‐Biolab, China), Histone H3 (1:6000, A17562, Abclonal, China), H3K18ac (1:1000, A7257, Abclonal, China), H4K5ac (1:1000, A19525, Abclonal, China) and Pan‐Kac (1:1500, #9441, CST, USA) at a temperature of 4°C. Finally, goat anti‐mouse (1:6000, AS003, Abclonal, China) and goat anti‐rabbit IgG (1:6000, AS014, Abclonal, China) secondary antibodies conjugated to a fluorescent tag were then added to the membranes. The levels of Histone H3 was used as the loading control of the histones. The Tanon 5300 (Shanghai, China) was used to visualize chemiluminescent signals and quantified using Image J software.

### 2.14 Statistical analysis

Statistical analysis was performed using SPSS version 20.0 (IBM, Armonk, NY, USA). Each of the studies was conducted a minimum of three times, and quantitative data are presented as mean ± SD. Analysis of comparison between two groups was performed using Student's *t*‐test. Two‐way anova was used to compare multiple groups. *p* < 0.05 indicated a statistically significant difference.

## 3 RESULTS

### 3.1 HKla involved in pathological modulation of ventricular remodelling

**FIGURE 1:**
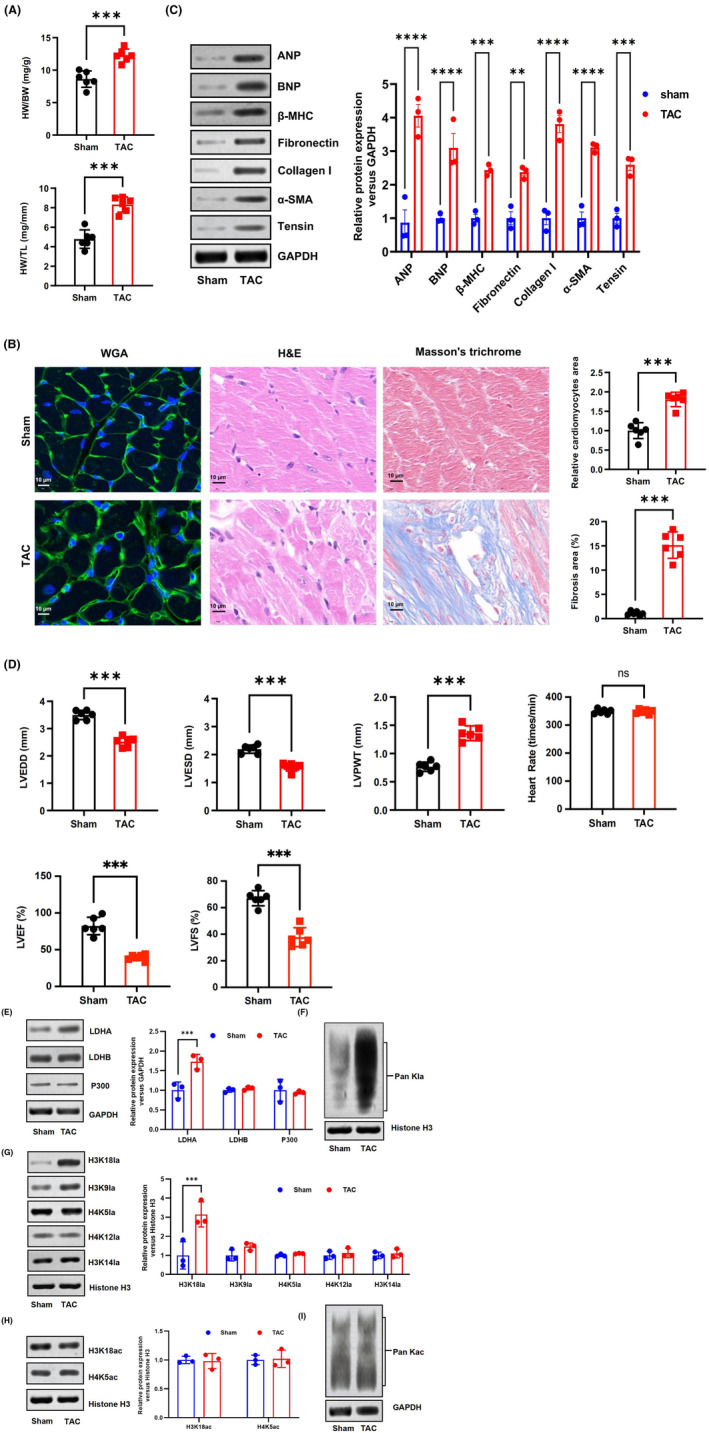
The expression of HKla is upregulated in cardiomyocytes of mice subjected to TAC. (A) The ratios of HW/BW and HW/TL were measured in both the sham and TAC groups (*n* = 6 mice/group). (B) Histological analysis of myocardial tissue. (C) Levels of markers for cardiac hypertrophy and fibrosis were measured in mice from the TAC and sham groups. (D) Echocardiography was performed to measure LVEDD, LVESD, LVPWT, heart rate, LVEF and LVFS in both the TAC and sham groups. (E) The levels of LDHA, LDHB and P300 were measured in the hearts of mice from the TAC and sham groups. (F, G) Western blot analysis was conducted to examine the levels of Pan Kla, H3K18la, H3K9la, H4K5la, H4K12la and H3K14la in hearts from mice in the TAC and sham groups. (H, I) The levels of H3K18ac, H4K5ac and pan Kac were measured in cardiomyocytes from both the TAC and sham groups (*n* = 3, **p < 0.01, ***p < 0.001, ****p < 0.0001, compared to the sham group).

To investigate the involvement of HKla in cardiac hypertrophy, we performed TAC surgery to establish a mouse model of cardiac hypertrophy. Next, we examined the expression of HKla in the TAC and sham groups and compared it with histone acetylation (HKac). Cardiac function was assessed using transthoracic echocardiography at 4 weeks after TAC surgery and compared between the TAC group and the sham group. The echocardiography results revealed an increase in LVPWT and a significant decrease in LVEDD, LVESD, LVEF and LVFS in the TAC group compared to the sham group (Figure 1D). Histological examination demonstrated that cardiac volume and mass were significantly greater in the TAC group compared to the sham group. The HW/BW and HW/TL were higher in the TAC group than in the sham group (Figure 1A). Subsequently, the analysis of left ventricular myocardial tissue showed that the cross‐sectional area of cardiomyocytes in the TAC group increased significantly, and the structural arrangement was disordered. The measurement of collagen abundance through Masson's trichrome staining. The study results indicate that the myocardial tissue in the sham group was mainly red, while the myocardial tissue in the TAC group showed an increase in blue collagen fibres, suggesting an aggravation of myocardial fibrosis (Figure 1B). Subsequently, the study analysed the myocardial injury situation and found that the levels of cardiac hypertrophy indicators ANP, BNP and β‐MHC were significantly upregulated in the TAC group compared to the sham group. Meanwhile, the expression of markers of interstitial fibrosis, including fibronectin, collagen I, α‐SMA and tensin, was upregulated in the TAC group compared to the sham group (Figure 1C). Cardiomyocyte hypertrophy and interstitial fibrosis are indicators of cardiac remodelling. These findings confirm that the TAC mouse model exhibits characteristic features of cardiac hypertrophy and dysfunction induced by pressure overload.

Furthermore, LDHA protein levels were significantly elevated in the TAC group compared to the control group (Figure 1E). To determine whether HKla is expressed in hypertrophic cardiomyopathy, the study performed western blot analysis using anti‐HKla and anti‐HKac antibodies separately on the TAC group and the sham group. Based on the results presented in Figure 1F, the TAC group exhibited increased levels of Pan Kla expression in cardiomyocytes compared to the sham group. Meanwhile, western blotting indicated that the expression of H3K18la was significantly increased (Figure 1G). In contrast, the HKac level remained unchanged, and there was no noticeable variation in the expression of Pan Kac, H3K18ac and H4K5ac between the sham and TAC groups (Figure 1H,I). Collectively, these data showed that HKla, particularly H3K18la, was overexpressed in the heart in a murine model of cardiac hypertrophy.

### 3.2 Lactate promotes HKla and aggravates cardiac hypertrophy

**FIGURE 2:**
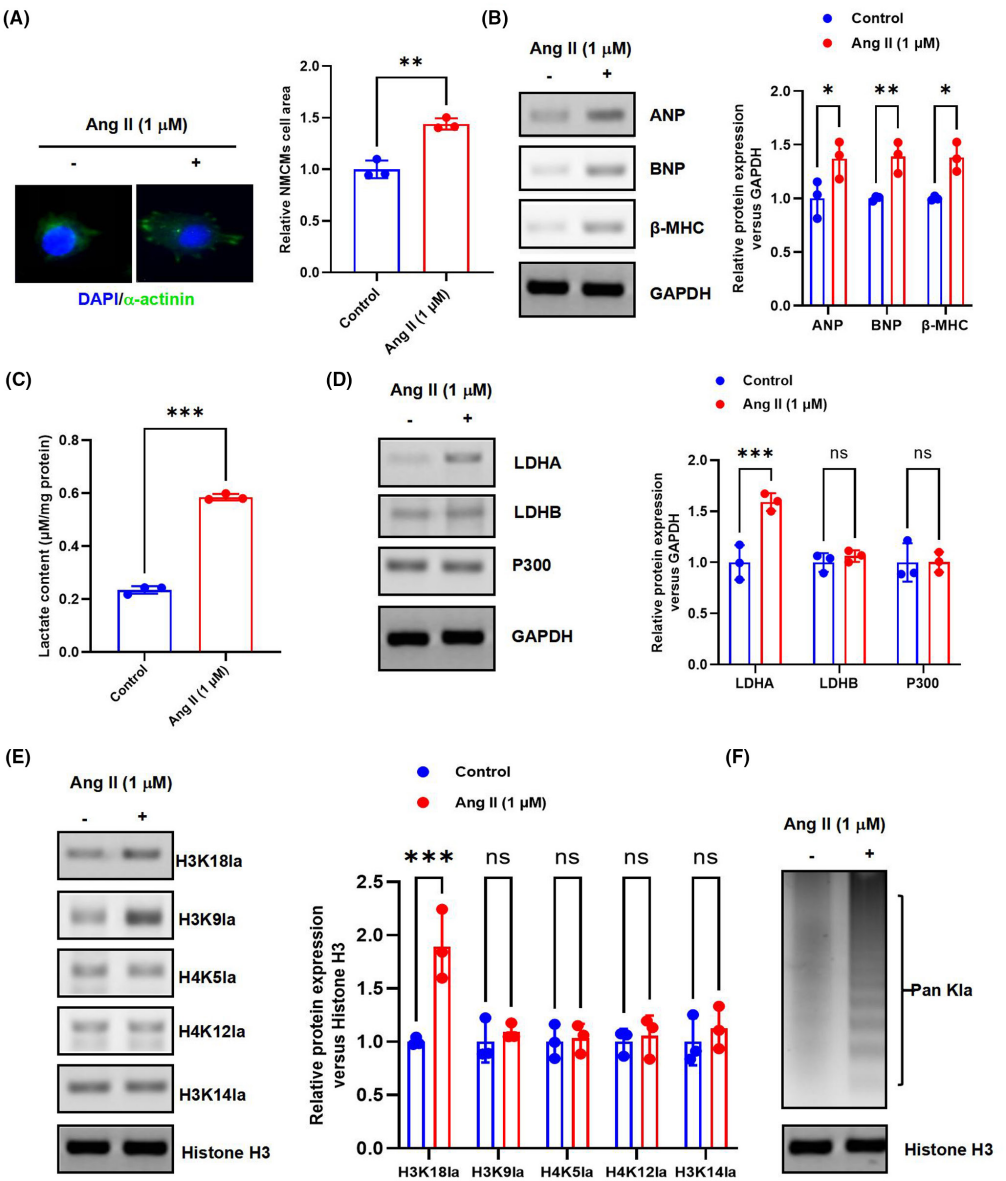
Ang II contributes to cardiomyocyte hypertrophy and promotes HKla. (A) After 24 h of Ang II stimulation, the cell surface area of NMCMs increased. (B) Representative western blots of ANP, BNP and β‐MHC in cultured NMCMs treated with Ang II (1 μM) for 24 h. (C) NMCMs were exposed to Ang II (1 μM) for 24 h, and intracellular lactate levels were evaluated using a colorimetric lactate measurement kit. (D) Expression levels of LDHA, LDHB and P300 proteins were detected in NMCMs using western blotting. (E, F) Western blot analysis was performed to measure the expression levels of H3K18la, H3K9la, H4K5la, H4K12la, H3K14la and Pan HKla in NMCMs (*n* = 3, *p < 0.05, **p < 0.01, ***p < 0.001, vs. control group).

**FIGURE 3:**
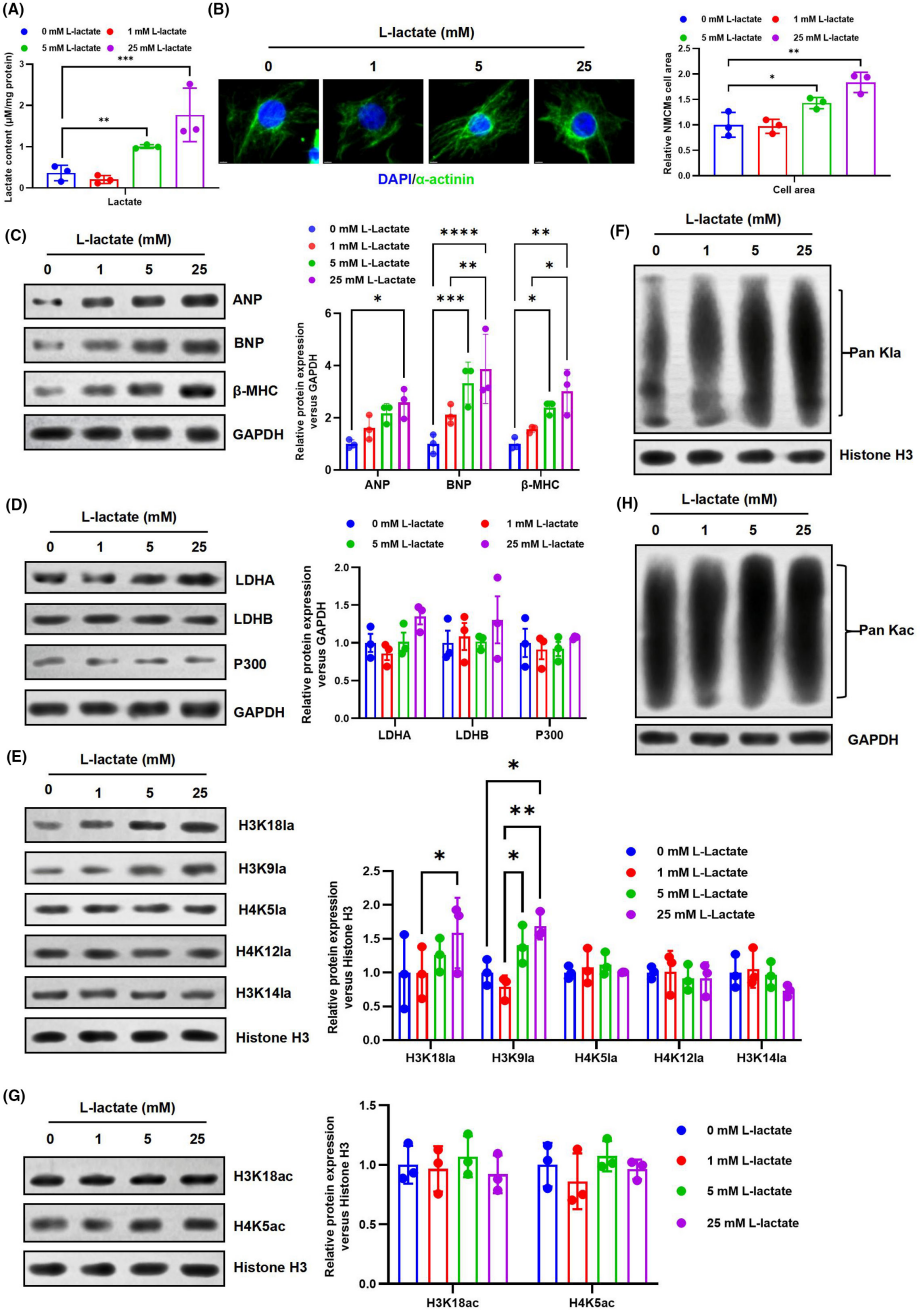
Lactate enhances HKla and exacerbates cardiac hypertrophy. (A) NMCMs were exposed to different concentrations of L‐lactate for 48 h, and intracellular lactate levels were evaluated using a colorimetric lactate measurement kit. (B) Effects of increasing lactate on the surface areas of cardiomyocytes. C‐H NMCMs were cultured with various concentrations of L‐lactate (0, 1, 5 and 10 mM) for 48 h. Afterward, cells at different concentrations were harvested for western blot analysis. (C) Effects of increasing lactate on the levels of cardiac hypertrophy markers. (D) Expression levels of LDHA, LDHB and P300 proteins were detected in NMCMs using western blotting. (E, F) Western blot analysis was performed to measure the expression levels of H3K18la, H3K9la, H4K5la, H4K12la, H3K14la and Pan HKla in NMCMs. (G, H) Expression levels of H3K18ac, H4K5ac and Pan Kac were detected in NMCMs using western blotting (*n* = 3, *p < 0.05, **p < 0.01, ***p < 0.001, ****p < 0.0001, vs. indicated group).

HKla is a novel histone mark that is stimulated by lactate and has been shown to correlate with glycolytic activity and lactate levels. It has also been found that HKla is L‐lactylated, rather than D‐lactylated.^22^ L‐lactate, a major product of glycolysis that moves between different tissues and cells, plays a crucial role in normal body function and disease processes.^5^ However, the role and manifestation of HKla in cardiac hypertrophy are still not well understood. To validate the regulatory effect of HKla on cardiac hypertrophy, the study established a model of cardiomyocyte hypertrophy by stimulating NMCMs with Ang II. First, analyse the effect of Ang II on HKla levels. The results showed that Ang II significantly increased cardiac hypertrophy and HKla levels in cardiomyocytes (Figure 2). Next, the study investigated the effect of exogenous lactate on HKla and cardiac hypertrophy, and incubated NMCMS with various concentrations of L‐lactate for 48 h. The results of the study showed that lactate levels in NMCMs increased continuously with increasing L‐lactate concentration (Figure 3A). Immunofluorescence was used to analyse the effect of exogenous lactate addition on cardiac hypertrophy. As shown in Figure 3B, the surface area of NMCMs increased with increasing L‐lactate concentration. Additionally, there was a significant increase in markers of cardiac hypertrophy, including ANP, BNP and β‐MHC (Figure 3C).

In order to further investigate the role of exogenous lactate in promoting hypertrophy through the upregulation of HKla expression, we utilized western blotting to analyse the expression of HKla and HKac in each treatment group of NMCMs models. Interestingly, the study revealed that lactate increased the level of HKla while having no effect on HKac. Western blotting demonstrated that increased levels of Pan Kla in NMCMs treated with exogenous lactate (Figure 3F). Consistent with our findings, Zhang et al. have previously demonstrated that HKla is influenced by glycolysis and lactate levels, and that L‐lactate serves as a regulator.^13^ Additionally, we observed that an increase in lactate levels was able to induce significant protein levels of H3K18la and H3K9la in the NMCMs (Figure 3E). Conversely, Pan Kac, H3K18ac and H4K5ac remained largely unchanged (Figure 3G,H). These results indicated that exogenous lactate increased intracellular lactate and HKla levels, and HKla overexpression aggravated Ang II‐induced NMCMs hypertrophy.

### 3.3 Glucose elevates lactate levels that could increase HKla expression and promote cardiac hypertrophy

**FIGURE 4:**
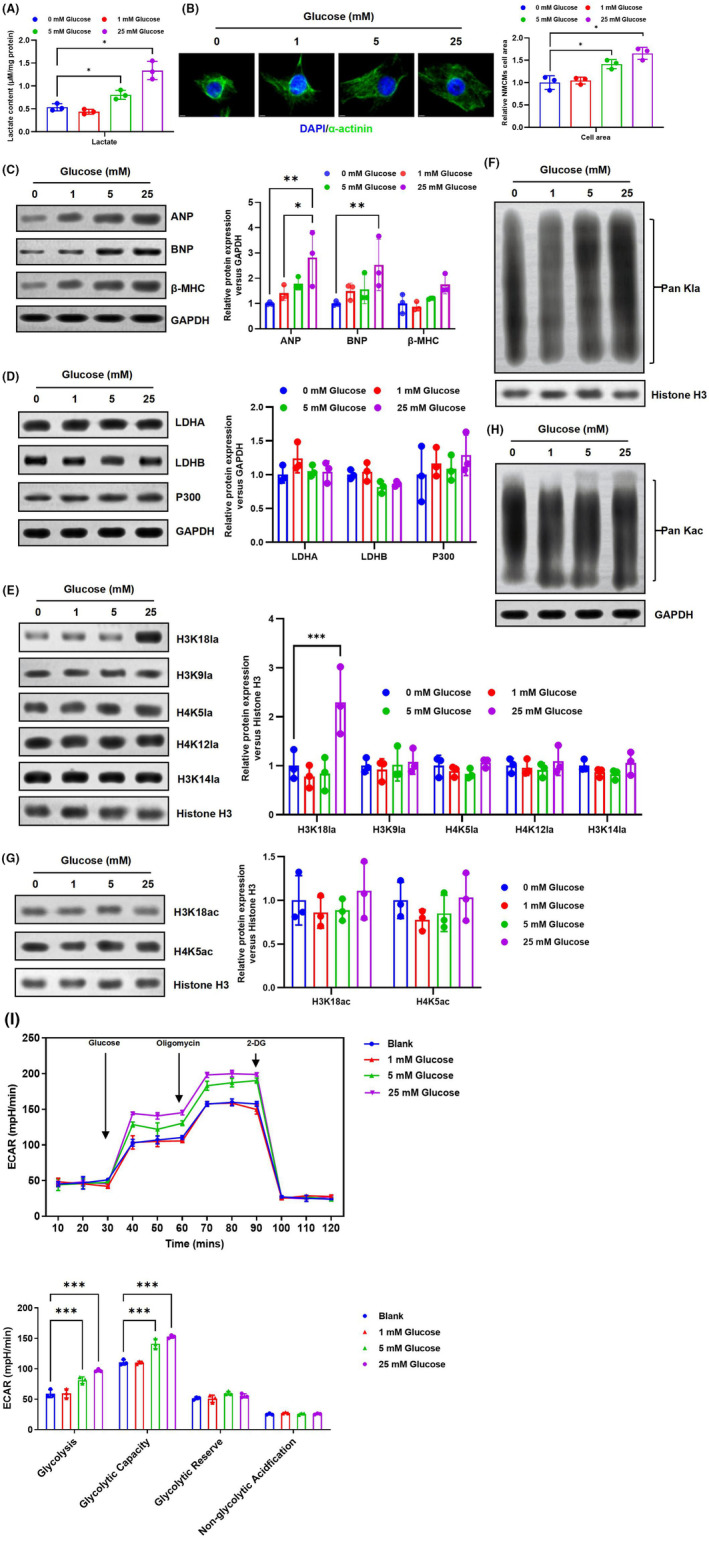
Glucose increases lactate levels, which upregulate HKla expression. (A) Lactate levels in NMCMs cultured with different concentrations of glucose were measured by a lactate colorimetric kit. (B) Effects of glucose increase on cardiomyocyte surface areas. (C‐H) NMCMs were cultured for 48 h with different concentrations of glucose (0, 1, 5 and 25 mM). Cells at varying concentrations were harvested and utilized for western blot analysis. (C) Effects of increasing glucose on the levels of cardiac hypertrophy markers. (D) LDHA, LDHB and P300 protein expression levels in NMCMs were assessed using western blotting. (E, F) Western blot analysis was performed to measure the expression levels of H3K18la, H3K9la, H4K5la, H4K12la, H3K14la and Pan HKla in NMCMs. (G, H) Expression levels of H3K18ac, H4K5ac and Pan HKla were detected in NMCMs using western blotting. (I) The glycolysis ability of cardiomyocytes was analysed by Seahorse ECAR assay (*n* = 3, *p < 0.05, **p < 0.01, ***p < 0.001, vs. indicated group).

Given that extracellular lactate can exacerbate cardiac hypertrophy by promoting HKla expression. We hypothesized that modulation of intracellular lactate production would also affect HKla levels. Glucose is initially converted through the glycolytic pathway to pyruvate, which can further be oxygenated in the mitochondria or converted to lactate.^23^ NMCMs were exposed to different concentrations of glucose for 48 h. The findings indicated that the induction of lactate production and glucose concentration was observed in a dose‐dependent manner, lactate level increased in NMCMs continuously with increasing glucose concentration (Figure 4A). Subsequently, immunofluorescence examination was used to evaluate the size of cardiomyocytes. The result showed that the surface area of cardiomyocytes increased with higher glucose concentration (Figure 4B). Moreover, the rise in intracellular lactate concentration can lead to impaired cardiac function, as demonstrated in Figure 4C. With the increase in extracellular glucose concentration, the expression of ANP and BNP, markers of myocardial hypertrophy in NMCMs, significantly increased. This suggests that elevated intracellular lactate concentrations can promote cardiac hypertrophy and impair cardiac function.

Previous studies have also demonstrated that changes in glucose metabolism kinetics and lactate levels affect the regulation of HKla.^24^ Subsequently, we further explored whether intracellular lactate could promote cardiac hypertrophy by increasing HKla expression. The results showed that HKla levels increased significantly with increasing glucose concentration. Western blot results showed a significant increase in the expression of Pan Kla and H3K18la with increasing intracellular lactate levels in NMCMs (Figure 4E,F). Previous research has shown that intracellular lactates can stimulate the promotion of histone H3 lactylation at the promoters of homeostatic genes, leading to their activation.^13^ However, HKac remained unchanged, and western blotting showed no significant changes in Pan Kac, H3K18ac and H4K5ac (Figure 4G,H). Consistently, Seahorse ECAR analysis also confirmed that glucose treatment significantly elevated the glycolysis ability in mouse cardiomyocytes (Figure 4I). These results suggest that as extracellular glucose concentration increased, cellular metabolic reprogramming and enhanced glycolysis led to increased lactate levels in NMCMs, which promoted H3K18la expression and exacerbated Ang II‐induced NMCM hypertrophy.

### 3.4 Inhibition of glucose metabolism may reduce HKla expression and alleviate myocardial hypertrophy

**FIGURE 5:**
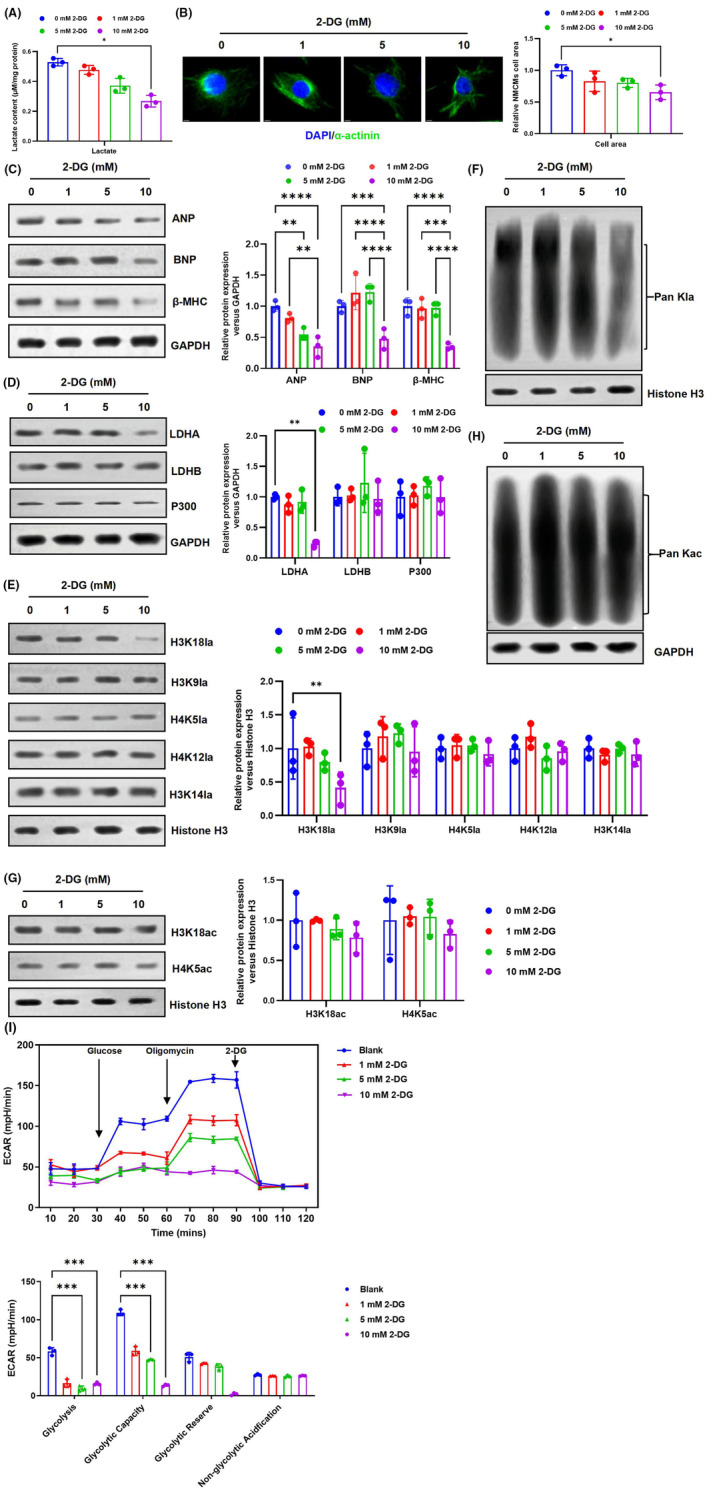
2‐DG can decrease HKla levels and inhibits the advancement of cardiac hypertrophy. (A) NMCMs were exposed to different concentrations of 2‐DG for 48 h, and intracellular lactate levels were evaluated using a colorimetric lactate measurement kit. (B) The impact of 2‐DG increase on the surface area of cardiomyocytes. (C–H) NMCMs were cultured with various concentrations of 2‐DG (0, 1, 5 and 10 mM) for 48 h, and cells were then collected for western blot analysis. (C) The impact of increasing 2‐DG on the levels of cardiac hypertrophy marker genes. (D) Western blot analysis detected the expression of LDHA, LDHB and P300 proteins in NMCMs. (E, F) Western blot analysis was conducted to examine the expression of H3K18la, H3K9la, H4K5la, H4K12la, H3K14la and Pan HKla in NMCMs. (G, H) Western blot analysis detected the expression of H3K18ac, H4K5ac and Pan Kac levels in NMCMs. (I) The glycolysis ability of cardiomyocytes was analysed by Seahorse ECAR assay (*n* = 3, *p < 0.05, **p < 0.01, ***p < 0.001, ****p < 0.0001, vs. indicated group).

Cells primarily obtain their energy through glucose metabolism, which involves the conversion of glucose into energy via the tricarboxylic acid (TCA) cycle and glycolysis. Dysregulation of the TCA cycle and glycolysis can cause reprogramming of cellular metabolism, leading to changes in the expression of HKla.^25^ The responsiveness of HKla to different environmental stimuli varies significantly. To investigate whether inhibiting lactate concentrations in cells can inhibit myocyte hypertrophy, we conducted a study by inhibiting glucose metabolism. The study treated cultured NMCMs with a glycolysis inhibitor, the non‐metabolizable glucose analogue 2‐deoxy‐D‐glucose (2‐DG) for 48 h. The results showed that increasing concentrations of 2‐DG led to a decrease in lactate production in NMCMs (Figure 5A). Simultaneously, at lower lactate concentrations, the expression of LDHA was significantly reduced (Figure 5D). To assess cardiomyocyte size, we performed immunofluorescence analysis of cell surface area. The results revealed that the surface area of hypertrophic cardiomyocytes decreased with increasing concentrations of 2‐DG, as depicted in Figure 5B. Additionally, the increase in 2‐DG concentration corresponded to a decrease in the protein levels of cardiac hypertrophy indicators ANP, BNP and β‐MHC (Figure 5C). These results suggest that inhibition of NMCMs lactate production may improve cardiac function.

To investigate the potential inhibitory effect of lactate on the progression of cardiac hypertrophy by downregulating HKla expression, we conducted western blot analysis to examine the levels of HKla and HKac in NMCMs treated with varying concentrations of 2‐DG. The findings indicated that increasing the concentration of the glycolysis inhibitor led to a reduction in lactate production, resulting in a decrease in HKla levels. Western blotting results demonstrated a significant decrease in the expression of Pan Kla, and H3K18la with increasing concentrations of 2‐DG (Figure 5E,F). In contrast, the levels of Pan Kac, H3K18ac and H4K5ac did not change significantly (Figure 5G,H). Consistently, Seahorse ECAR analysis further confirmed that 2‐DG obviously inhibited the glycolysis (Figure 5I). These results further support the notion that glucose metabolism plays a crucial role in the regulation of HKla and gene expression.^13^ These findings suggest that increasing the concentration of 2‐DG can inhibit glucose metabolism, leading to a decrease in lactate levels, accompanied by a reduction in HKla levels, ultimately suppressing cardiomyocyte hypertrophy.

### 3.5 Suppressing LDH function reduces HKla levels and inhibits the development of myocardial hypertrophy

**FIGURE 6:**
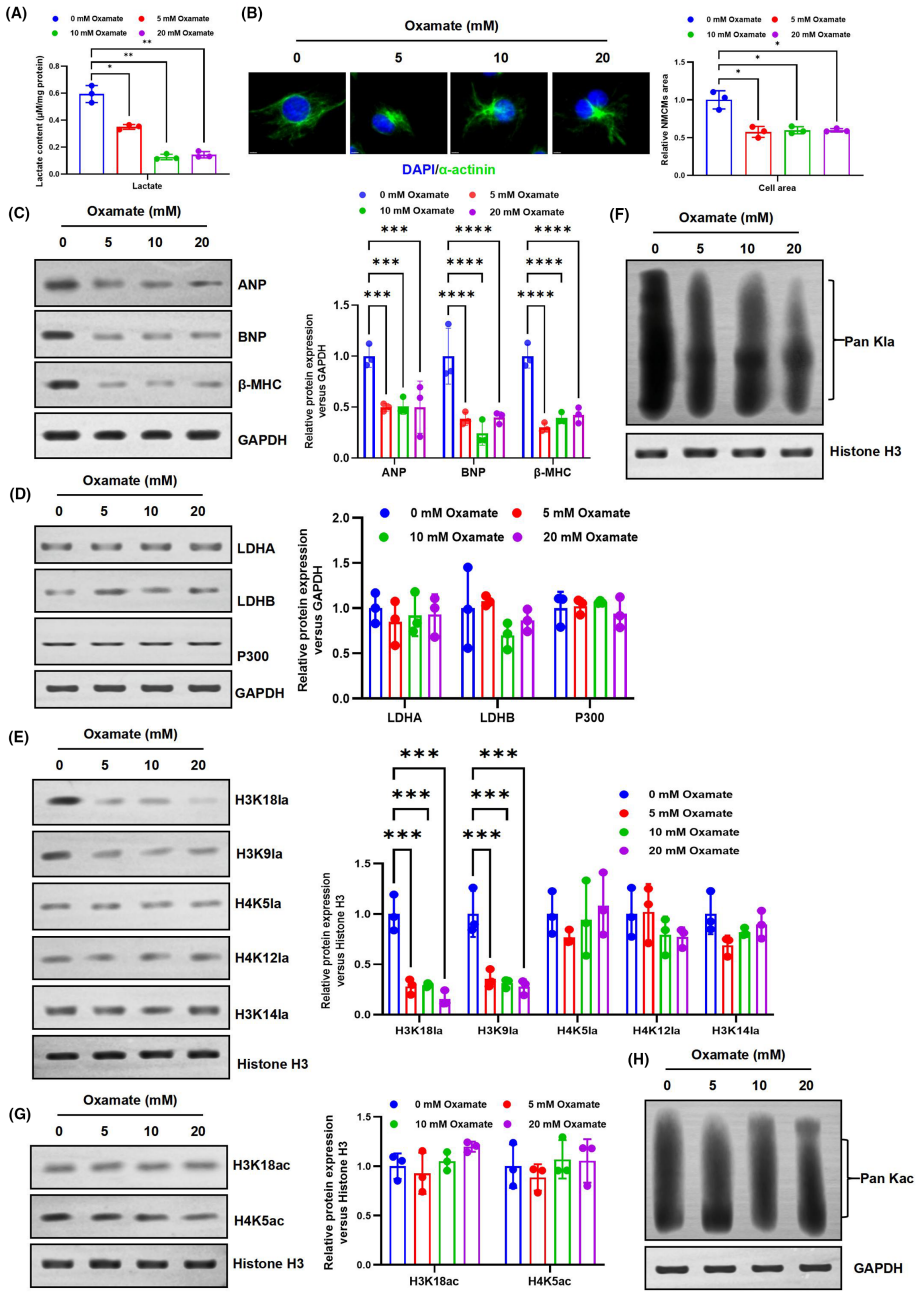
Oxamate can reduce HKla levels and inhibit the progression of cardiac hypertrophy. (A) Lactate levels in NMCM cultured with different concentrations of oxamate were measured by a lactate colorimetric kit. (B) The effect of increased oxamate concentration on the surface area of cardiomyocytes was detected. (C–H) The NMCMs model was cultured for 48 h using different concentrations of oxamate (0, 5, 10 and 20 mM), and cells were then collected for western blot analysis. (C) The impact of an increase in oxalate concentration on the levels of marker genes for cardiac hypertrophy. (D) The protein expression levels of LDHA, LDHB and P300 in NMCMs exposed to different concentrations of oxamate were analysed by western blotting. (E and F) Western blot analysis was performed to assess the expression level of H3K18la, H3K9la, H4K5la, H4K12la, H3K14la and Pan HKla in NMCMs. (G and H) Expression of H3K18ac, H4K5ac and Pan Kac levels were detected in NMCMs by western blotting (*n* = 3, *p < 0.05, **p < 0.01, ***p < 0.001, ****p < 0.0001, vs. indicated group).

The level of lactate is determined by the balance between the TCA cycle and glycolysis. A molecule of glucose can produce two molecules of ATP and pyruvate without consuming oxygen. The pyruvate is then converted into lactate by cytosolic lactate dehydrogenases (LDHs).^26^ To investigate the correlation between the lactate level and HKla, we suppressed LDH function using oxamate, a potent and selective inhibitor that effectively decreases lactate production during glycolysis. Initially, the NMCMs model was incubated with different concentrations of oxamate (0, 5, 10 and 20 mmol/L) for 48 h. As expected, the expression of lactate levels was downregulated with an increase in oxamate concentration (Figure 6A). Subsequently, we used immunofluorescence analysis of cell surface area to assess cardiomyocyte size. As shown in Figure 6B, the surface area of hypertrophic cardiomyocytes decreased with increasing oxamate concentration. Furthermore, the expression of cardiac hypertrophy markers ANP, BNP and β‐MHC decreased with increasing oxamate concentration (Figure 6C). These results suggest that the LDH inhibitor oxamate attenuates the progression of Ang II‐induced hypertrophy in NMCMs.

In order to investigate the impact of changes in cellular metabolism and lactate levels on HKla, western blotting was utilized to assess the expression of HKla and HKac in NMCMs treated with varying concentrations of oxamate. The findings indicate that the suppression of LDH activity effectively inhibits lactate and HKla levels in NMCMs. Western blotting demonstrated that as the concentration of oxamate increased, there was a significant decrease in Pan Kla levels, as well as reductions in H3K18la and H3K9la (Figure 6E,F). However, no significant changes were observed in the level of HKac, including Pan Kac, H3K18ac and H4K5ac (Figure 6G,H). Collectively, these results suggest that inhibiting LDH activity reduces HKla levels, thereby mitigating hypertrophy in NMCMs.

### 3.6 Inhibition of LDHA activity could reduce HKla and limit myocardial hypertrophy

**FIGURE 7:**
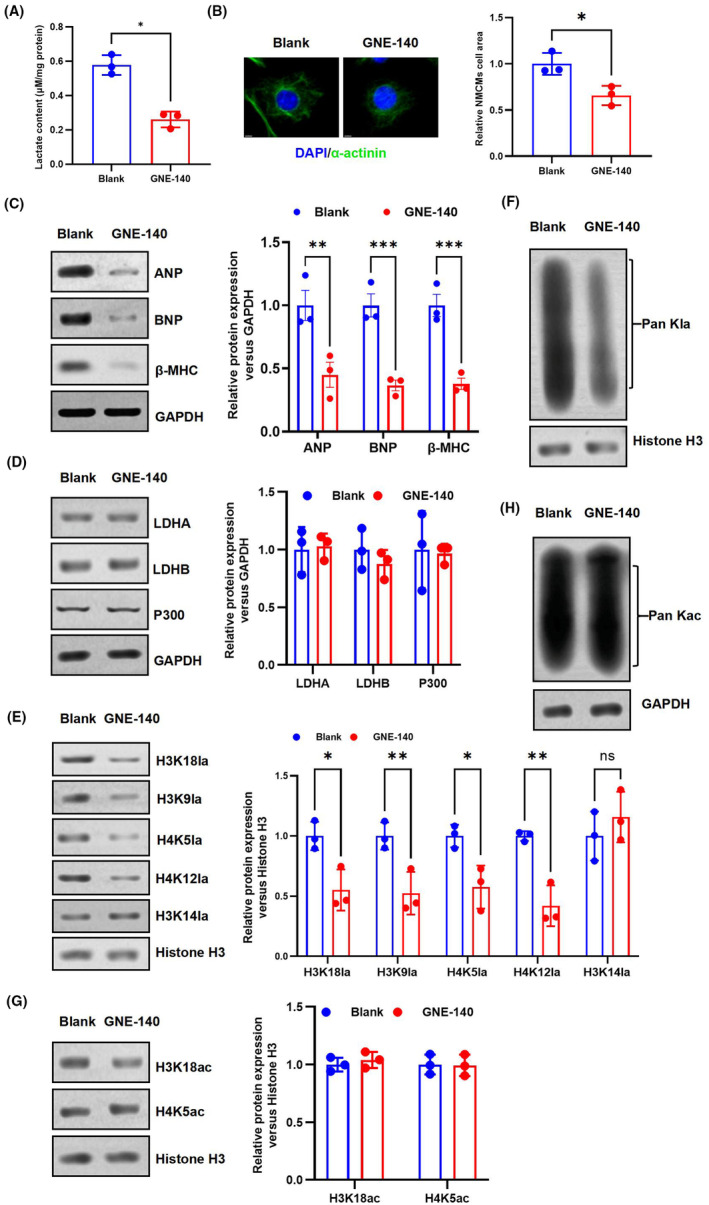
GNE‐140 may reduce HKla levels and inhibit the progression of cardiac hypertrophy. (A) Comparison of intracellular lactate levels between the GNE‐140 group and the blank group. (B) The effects of GNE‐140 on the surface areas of NMCMs compared to the blank group. (C–H) NMCMs were treated with GNE‐140 (10 μM) for 48 h. For western blot analysis, cells were collected from both the GNE‐140 and blank groups. (C) The levels of cardiac hypertrophy markers between the GNE‐140 group and the control group. (D) The expression levels of LDHA, LDHB and P300 proteins were detected in the control and GNE‐140 groups by western blotting. (E, F) Western blot analysis was performed to assess the expression levels of H3K18la, H3K9la, H4K5la, H4K12la, H3K14la and Pan Hkla in the control and GNE‐140 groups. (G, H) Western blotting was used to measure the levels of H3K18ac, H4K5ac and Pan Kac in the control and GNE‐140 groups (*n* = 3, *p < 0.05, **p < 0.01, ***p < 0.001, vs. blank group).

The conversion of pyruvate into lactate is catalysed by LDHA, which serves as the key enzyme.^27^ Furthermore, the activity of LDHA also plays a significant role in the synthesis of acetyl‐CoA.^13^ To further investigate the impact of inhibiting lactate synthesis on HKla expression, hypertrophied NMCMs were treated with GNE‐140, a specific inhibitor of LDHA, to assess its influence on HKla expression. The results demonstrated a reduction in lactate levels in NMCMs after 48 h of treatment with GNE‐140 (10 μM) compared to the control group (Figure 7A). Next, immunofluorescence was employed to evaluate the dimensions of cardiomyocytes. As shown in Figure 7B, the surface area of cardiomyocytes in the GNE‐140 group was reduced compared to that in the control group. Additionally, the GNE‐140 group exhibited a significant reduction in the levels of myocardial hypertrophy markers ANP, BNP and β‐MHC compared to the blank group (Figure 7C). These results suggest that the LDHA inhibitor GNE‐140 attenuates the progression of Ang II‐induced NMCM hypertrophy.

To further investigate whether inhibited LDHA activity reduces HKla expression, we detected HKla expression in the GNE‐140 group and the blank group by western blotting, and compared with the level of HKac. The findings indicated that the levels of Pan‐Kla, H3K18la, H3K9la, H4K5la and H4K12la were significantly reduced in the GNE‐140 group compared to the control group (Figure 7E,F). This indicates that inhibition of LDHA activity effectively suppresses the levels of lactate and HKla in NMCMs. Zhang et al. have also shown that during M1 polarization of LDHA‐deficient macrophages, both lactate production and global HKla levels are reduced.^13^ However, the HKac level remained unchanged, GNE‐140 had no significant effect on Pan Kac, H3K18ac and H4K5ac expression levels in NMCMs treated with AngII stimulation, compared with the blank group (Figure 7G,H). These results suggest that inhibition of lactate levels and HKla expression by inhibiting LDHA activity inhibits cardiomyocyte hypertrophy.

## 4 DISCUSSION

Cardiovascular disease (CVD) is a significant global health threat. Despite advancements in primary prevention, the incidence of CVD has continued to rise in recent years. Myocardial hypertrophy can be caused by various cardiovascular diseases, including coronary heart disease, hypertension, valvular disease and congenital heart disease, when they progress to a certain extent. The pathophysiological process of cardiac hypertrophy is intricate, involving multiple factors and signalling pathways within cardiomyocytes.^28^ During cardiac hypertrophy, various intracellular cascades are activated, including oxidative stress, mitochondrial damage, ionic changes and excessive activation of embryonic genes.^29^ The continuous increase in pressure load leads to ventricular remodelling, which can ultimately result in HF.

Eukaryotes produce significant amounts of lactate during glycolysis.^30^ Lactate is significantly produced during hypoxia and the Warburg phenomenon, both of which are connected to a variety of cellular activities and diseases.^31^ The cardiomyocyte is a unique muscle cell type that exhibits rhythmic contractile function. Glucose metabolism is vital for maintaining physiological heart function, and in times of high demand, cardiomyocytes gradually shift to using lactate as an energy source. In cases of cardiac hypertrophy and hypoxia, there is an alteration in cardiac metabolism, leading to increased glucolysis and the production of large amounts of lactate. Recent studies have shown that lactate plays a key role in a variety of physiological and pathological processes through energy regulation, REDOX buffering and metabolic regulation, as an active signalling molecule.^32^ Additionally, study has suggested that lactate plays an important role in reprogramming the epigenome, as intracellular lactate can induce epigenetic modifications by inhibiting lactylation of histone lysine residues in macrophages in an NAD(+)‐independent manner.^33^ The relationship between glucose metabolism and cardiovascular disease is important for prevention and treatment.

Cardiomyocyte enlargement is the underlying pathological mechanism of cardiac hypertrophy and is a common pathological change in various types of HF. The occurrence of damage to the heart muscle initiates a series of communication pathways that activate the production of genes associated with hypertrophy at various stages.^34^ Studies have shown that myocardial tissue fragility caused by cardiac amyloidosis increases the myocardium's susceptibility to damage.^35^ In addition, The hearts of patients with the desmoglin‐2 R119X mutation show abnormal electrical activity and structural fragility, resulting in marked enlargement of both biventricles and decreased cardiac systolic function.^36^ Myocardial amyloidosis increases myocardial structural fragility and damage, leading to ventricular enlargement and decreased cardiac function.

Studies have shown that epigenetics and epitranscriptome are involved in the pathogenesis of pathological cardiac hypertrophy.^37^ By regulating gene transcription, HKla, a novel epigenetic modification, plays a critical role in maintaining various physiological conditions and triggering numerous pathological mechanisms.^38^ In terms of transcription and antigenic variation, The expression or repression of chromatin is determined by the PTM status of the core histones.^14^ Zhang et al. identified 28 lactylation sites, including H3, H4, H2A and H2B.^13^ Meanwhile, it has been shown that HKla sites on H3 and H4 are located at the N‐ and C‐terminal ends and include numerous PTM sites with different modifications.^39^ Zhang et al. found 142 lactamated proteins and 483 lactamated sites in Ang II‐induced mouse HF models.^40^

This study identified the key role of HKla in pathological cardiac hypertrophy, and suggests it could be a potential treatment target. Following TAC surgery of C57BL/6 mice, there was a significant increase in cardiomyocyte size, amount of fibrosis/collagen and the presence of cardiac hypertrophic markers. There are multiple methods for euthanizing mice, with end‐diastolic cardiac arrest representing the optimal condition for measuring cardiomyocyte size in mice. However, CO₂ inhalation does not entirely guarantee end‐diastolic cardiac arrest. Therefore, there are certain limitations. Subsequently, we examined the dynamics of HKla, in comparison with HKac. In the cardiac tissues of TAC mice, we found the HKla was overexpressed, the expression levels of Pan Kla and H3K18la were upregulated, whereas those of HKac were not significantly altered. The identification of HKla‐related molecules in our investigation could offer valuable insights and a mechanistic foundation for the amelioration of cardiac hypertrophy.

Next, the model of cardiomyocyte hypertrophy was established by administering Ang II to NMCMs. The addition of exogenous glucose and L‐lactate increased lactate concentration and HKla levels in NMCMs. Western blotting revealed a significant increase in H3K18la expression, thereby promoting cardiomyocyte hypertrophy. Galle et al. has also detected that H3K18la was the promoters of the highest expressed genes.^41^ In contrast, glycolysis inhibitors (2‐DG), LDH inhibitors (oxamate) and LDHA inhibitors (GNE‐140) were observed to reduce lactate production and HKla expression in NMCMs. Western blotting demonstrated a marked decrease in H3K18la levels, thus inhibiting cardiomyocyte hypertrophy. In vitro studies further demonstrated that overexpression of HKla exacerbated Ang II‐induced hypertrophy in NMCMs, while inhibition of HKla resulted in the opposite phenotype.

Studies have shown that lactate plays a critical role as an active signalling molecule in several physiological and pathological processes.^32^ Meanwhile, lactate plays a crucial role in modifying and regulating gene expression in HKla. Targeting lactate metabolism is emerging as a promising and effective therapeutic strategy. Our research has revealed a new effect of H3K18la on cardiac hypertrophy, thereby enhancing our cognition of the HKla modification. Preventing cardiac hypertrophy from occurring and developing is beneficial in reducing HF incidence and mortality. Based on these findings, H3K18la could be a potential therapeutic target for treating pathological cardiac hypertrophy.

## 5 CONCLUSIONS

The present study demonstrates that HKla is involved in the pathological process of cardiac hypertrophy, especially H3K18la, whose increase/decrease is associated with changes in cardiac hypertrophy. The targeting of lactate metabolism is emerging as a valuable therapeutic approach. Research on HKla epigenetic control of genes is just beginning. Consequently, it is expected that the study of lactate metabolism will lead to the development of new specific drugs that can effectively control cellular functions and minimize side effects.

## 6 LIMITATION

A limitation of this study is the lack of detection of lactylation in non‐histone proteins. Furthermore, apart from histone H3, an increasing number of non‐histone proteins participate in various pathophysiological processes. The lactylation of histones might also be regulated by additional key enzymes in cellular metabolism. These need to be further investigated.

## AUTHOR CONTRIBUTIONS

**Shuai‐Shuai Zhao:** Data curation (equal); formal analysis (equal); writing – original draft (equal); writing – review and editing (equal). **Jinlong Liu:** Data curation (equal); formal analysis (equal); methodology (equal); supervision (equal); writing – review and editing (equal). **Qi‐Cai Wu:** Funding acquisition (equal); investigation (equal); project administration (equal); resources (equal); validation (equal). **Xue‐Liang Zhou:** Conceptualization (equal); funding acquisition (equal); project administration (equal); resources (equal); validation (equal); visualization (equal).

## FUNDING INFORMATION

This work was supported by grants from the National Natural Science Foundation of China (nos: 82070303, 81970199, 82360060 and 82160082) and the Natural Science Foundation of Jiangxi Province (nos: 20232ACB206003, 20232BAB206113 and 20202BAB206006).

## CONFLICT OF INTEREST STATEMENT

The authors declare no conflicts of interest.

## Data Availability

Data and materials supporting the findings of this study are available from the corresponding author upon reasonable request.

## References

[1] Oldfield CJ , Duhamel TA , Dhalla NS . Mechanisms for the transition from physiological to pathological cardiac hypertrophy. Can J Physiol Pharmacol. 2020;98:74‐84. 31815523 10.1139/cjpp-2019-0566

[2] Parbhudayal RY , Harms HJ , Michels M , van Rossum AC , Germans T , van der Velden J . Increased myocardial oxygen consumption precedes contractile dysfunction in hypertrophic cardiomyopathy caused by pathogenic TNNT2 gene variants. J Am Heart Assoc. 2020;9:e015316. 32290750 10.1161/JAHA.119.015316 PMC7428531

[3] You J , Wu J , Zhang Q , et al. Differential cardiac hypertrophy and signaling pathways in pressure versus volume overload. Am J Physiol Heart Circ Physiol. 2018;314:H552‐H562. 29196344 10.1152/ajpheart.00212.2017

[4] Ussher JR , Wang W , Gandhi M , et al. Stimulation of glucose oxidation protects against acute myocardial infarction and reperfusion injury. Cardiovasc Res. 2012;94:359‐369. 22436846 10.1093/cvr/cvs129

[5] Rabinowitz JD , Enerback S . Lactate: the ugly duckling of energy metabolism. Nat Metab. 2020;2:566‐571. 32694798 10.1038/s42255-020-0243-4 PMC7983055

[6] Ippolito L , Morandi A , Giannoni E , Chiarugi P . Lactate: a metabolic driver in the tumour landscape. Trends Biochem Sci. 2019;44:153‐166. 30473428 10.1016/j.tibs.2018.10.011

[7] Bhagat TD , Von Ahrens D , Dawlaty M , et al. Lactate‐mediated epigenetic reprogramming regulates formation of human pancreatic cancer‐associated fibroblasts. elife. 2019;8:e50663. 31663852 10.7554/eLife.50663 PMC6874475

[8] Souto‐Carneiro MM , Klika KD , Abreu MT , et al. Effect of increased lactate dehydrogenase a activity and aerobic glycolysis on the proinflammatory profile of autoimmune CD8+ T cells in rheumatoid arthritis. Arthritis Rheumatol. 2020;72:2050‐2064. 32602217 10.1002/art.41420

[9] Rosca MG , Tandler B , Hoppel CL . Mitochondria in cardiac hypertrophy and heart failure. J Mol Cell Cardiol. 2013;55:31‐41. 22982369 10.1016/j.yjmcc.2012.09.002 PMC3805050

[10] Luna RCP , de Oliveira Y , Lisboa JVC , et al. Insights on the epigenetic mechanisms underlying pulmonary arterial hypertension. Braz J Med Biol Res. 2018;51:e7437. 30365723 10.1590/1414-431X20187437 PMC6207290

[11] Vasconcelos ESJ , Simao D , Terrasso AP , et al. Unveiling dynamic metabolic signatures in human induced pluripotent and neural stem cells. PLoS Comput Biol. 2020;16:e1007780. 32298259 10.1371/journal.pcbi.1007780 PMC7188302

[12] Notarangelo G , Haigis MC . Sweet temptation: from sugar metabolism to gene regulation. Immunity. 2019;51:980‐981. 31851904 10.1016/j.immuni.2019.11.008

[13] Zhang D , Tang Z , Huang H , et al. Metabolic regulation of gene expression by histone lactylation. Nature. 2019;574:575‐580. 31645732 10.1038/s41586-019-1678-1 PMC6818755

[14] Stillman B . Histone modifications: insights into their influence on gene expression. Cell. 2018;175:6‐9. 30217360 10.1016/j.cell.2018.08.032

[15] Zhang N , Jiang N , Yu L , et al. Protein lactylation critically regulates energy metabolism in the protozoan parasite *Trypanosoma brucei* . Front Cell Dev Biol. 2021;9:719720. 34722503 10.3389/fcell.2021.719720 PMC8551762

[16] Raghow R . An ‘Omics’ perspective on cardiomyopathies and heart failure. Trends Mol Med. 2016;22:813‐827. 27499035 10.1016/j.molmed.2016.07.007

[17] Li J , Yan C , Wang Y , et al. GCN5‐mediated regulation of pathological cardiac hypertrophy via activation of the TAK1‐JNK/p38 signaling pathway. Cell Death Dis. 2022;13:421. 35490166 10.1038/s41419-022-04881-y PMC9056507

[18] Liu MY , Yue LJ , Luo YC , et al. SUMOylation of SIRT1 activating PGC‐1alpha/PPARalpha pathway mediates the protective effect of LncRNA‐MHRT in cardiac hypertrophy. Eur J Pharmacol. 2022;930:175155. 35863508 10.1016/j.ejphar.2022.175155

[19] Jiang DS , Liu Y , Zhou H , et al. Interferon regulatory factor 7 functions as a novel negative regulator of pathological cardiac hypertrophy. Hypertension. 2014;63:713‐722. 24396025 10.1161/HYPERTENSIONAHA.113.02653 PMC5349187

[20] Wang J , Yang P , Yu T , et al. Lactylation of PKM2 suppresses inflammatory metabolic adaptation in pro‐inflammatory macrophages. Int J Biol Sci. 2022;18:6210‐6225. 36439872 10.7150/ijbs.75434 PMC9682528

[21] Kawamura T , Ono K , Morimoto T , et al. Acetylation of GATA‐4 is involved in the differentiation of embryonic stem cells into cardiac myocytes. J Biol Chem. 2005;280:19682‐19688. 15764815 10.1074/jbc.M412428200

[22] Moreno‐Yruela C , Zhang D , Wei W , et al. Class I histone deacetylases (HDAC1‐3) are histone lysine delactylases. Sci Adv. 2022;8:eabi6696. 35044827 10.1126/sciadv.abi6696 PMC8769552

[23] Potente M , Carmeliet P . The link between angiogenesis and endothelial metabolism. Annu Rev Physiol. 2017;79:43‐66. 27992732 10.1146/annurev-physiol-021115-105134

[24] Varner EL , Trefely S , Bartee D , et al. Quantification of lactoyl‐CoA (lactyl‐CoA) by liquid chromatography mass spectrometry in mammalian cells and tissues. Open Biol. 2020;10:200187. 32961073 10.1098/rsob.200187 PMC7536085

[25] Liberti MV , Locasale JW . Histone lactylation: a new role for glucose metabolism. Trends Biochem Sci. 2020;45:179‐182. 31901298 10.1016/j.tibs.2019.12.004

[26] Ma LN , Huang XB , Muyayalo KP , Mor G , Liao AH . Lactic acid: a novel signaling molecule in early pregnancy? Front Immunol. 2020;11:279. 32180770 10.3389/fimmu.2020.00279 PMC7057764

[27] Lee TY . Lactate: a multifunctional signaling molecule. Yeungnam Univ J Med. 2021;38:183‐193. 33596629 10.12701/yujm.2020.00892 PMC8225492

[28] Romero‐Becerra R , Santamans AM , Folgueira C , Sabio G . p38 MAPK pathway in the heart: new insights in health and disease. Int J Mol Sci. 2020;21(19):7412. 33049962 10.3390/ijms21197412 PMC7582802

[29] Facundo HT , Brainard RE , Watson LJ , et al. O‐GlcNAc signaling is essential for NFAT‐mediated transcriptional reprogramming during cardiomyocyte hypertrophy. Am J Physiol Heart Circ Physiol. 2012;302:H2122‐H2130. 22408028 10.1152/ajpheart.00775.2011 PMC3362113

[30] Izzo LT , Wellen KE . Histone lactylation links metabolism and gene regulation. Nature. 2019;574:492‐493. 31645737 10.1038/d41586-019-03122-1

[31] Hui S , Ghergurovich JM , Morscher RJ , et al. Glucose feeds the TCA cycle via circulating lactate. Nature. 2017;551:115‐118. 29045397 10.1038/nature24057 PMC5898814

[32] Brooks GA . Lactate as a fulcrum of metabolism. Redox Biol. 2020;35: 101454. 32113910 10.1016/j.redox.2020.101454 PMC7284908

[33] Chen L , Huang L , Gu Y , Cang W , Sun P , Xiang Y . Lactate‐lactylation hands between metabolic reprogramming and immunosuppression. Int J Mol Sci. 2022;23(19):11943. 36233246 10.3390/ijms231911943 PMC9569569

[34] Huang CK , Kafert‐Kasting S , Thum T . Preclinical and clinical development of noncoding RNA therapeutics for cardiovascular disease. Circ Res. 2020;126:663‐678. 32105576 10.1161/CIRCRESAHA.119.315856 PMC7043728

[35] Terashima R , Nakagawa T , Hara H , Hiroi Y . Left atrial intramural haematoma: a fatal complication of cardiac amyloidosis—a case report. Eur Heart J Case Rep. 2023;7:ytad116. 36969510 10.1093/ehjcr/ytad116 PMC10035636

[36] Shiba M , Higo S , Kondo T , et al. Phenotypic recapitulation and correction of desmoglein‐2‐deficient cardiomyopathy using human‐induced pluripotent stem cell‐derived cardiomyocytes. Hum Mol Genet. 2021;30:1384‐1397. 33949662 10.1093/hmg/ddab127 PMC8283207

[37] Berulava T , Buchholz E , Elerdashvili V , et al. Changes in m6A RNA methylation contribute to heart failure progression by modulating translation. Eur J Heart Fail. 2020;22:54‐66. 31849158 10.1002/ejhf.1672

[38] Yu J , Chai P , Xie M , et al. Histone lactylation drives oncogenesis by facilitating m(6)A reader protein YTHDF2 expression in ocular melanoma. Genome Biol. 2021;22:85. 33726814 10.1186/s13059-021-02308-z PMC7962360

[39] Saha S . Histone modifications and other facets of epigenetic regulation in trypanosomatids: leaving their mark. MBio. 2020;11(5):e01079‐20. 32873754 10.1128/mBio.01079-20 PMC7468196

[40] Zhang N , Zhang Y , Xu J , et al. Alpha‐myosin heavy chain lactylation maintains sarcomeric structure and function and alleviates the development of heart failure. Cell Res. 2023;33:679‐698. 37443257 10.1038/s41422-023-00844-w PMC10474270

[41] Galle E , Wong CW , Ghosh A , et al. H3K18 lactylation marks tissue‐specific active enhancers. Genome Biol. 2022;23:207. 36192798 10.1186/s13059-022-02775-y PMC9531456

